# Stable First- and Last-Entry Behaviour into the Milking Parlour Is Associated with Temperament and Udder Health Indicators in Lacaune Dairy Ewes

**DOI:** 10.3390/ani16121868

**Published:** 2026-06-17

**Authors:** Ferenc Pajor, Krisztina Márta, Lilla Sándorová, Péter Póti, István Egerszegi, Ákos Bodnár

**Affiliations:** 1 Department of Animal Technology and Animal Welfare, Institute of Animal Sciences, Hungarian University of Agriculture and Life Sciences, H-2100 Gödöllő, Hungary; marta.krysztyna@gmail.com (K.M.); poti.peter@uni-mate.hu (P.P.); egerszegi.istvan@uni-mate.hu (I.E.); bodnar.akos@uni-mate.hu (Á.B.); 2 Department of Genetics and Genomics, Institute of Genetics and Biotechnology, Hungarian University of Agriculture and Life Sciences, H-2100 Gödöllő, Hungary; sandorova.lilla@uni-mate.hu

**Keywords:** entry order, dairy sheep, milking behaviour, milk yield, udder health, fatty acid profile, animal behaviour

## Abstract

This study evaluated whether stable first- and last-entry behaviour into the milking parlour is associated with behavioural traits, milk production, udder health, and milk quality in Lacaune dairy ewes. A total of 246 ewes were monitored during lactation, and animals showing highly consistent first- or last-entry behaviour across six observations were selected. The final dataset included 18 first-entry ewes (FG) and 23 last-entry ewes (LG). FG ewes were heavier than LG ewes (62.11 ± 5.25 vs. 56.83 ± 4.94 kg; *p* = 0.002) and had higher temperament scores, indicating calmer behaviour (4.00 ± 1.08 vs. 2.96 ± 1.26; *p* = 0.011). In unadjusted comparisons, FG ewes showed higher daily milk yield (1.20 vs. 1.04 kg/day), lactation milk yield (200.31 vs. 147.30 kg), and longer lactation length (167.78 vs. 143.96 days; *p* < 0.05). After adjustment for body weight, age, parity, and temperament, these differences were no longer significant, suggesting that they mainly reflected animal-level characteristics. Among udder health indicators, electrical conductivity remained lower in FG ewes after adjustment (3.72 ± 0.08 vs. 4.03 ± 0.07 mS/cm; *p* = 0.012), whereas log_10_-transformed somatic cell count (LSCC) differed only numerically. FG milk samples also showed fewer udder pathogen-positive results than LG samples (4.6% vs. 16.7%; *p* = 0.012), although these findings should be interpreted as exploratory sample-level associations. Milk fat and protein percentages did not differ between groups. Fatty acid differences were nominally significant but not after multiple-testing correction. Overall, stable entry behaviour reflected temperament and selected udder health indicators, whereas its relationship with milk production was mainly explained by animal-level characteristics.

## Simple Summary

In dairy sheep farming, the order in which animals enter the milking parlour may reflect their behaviour, temperament, and social status. This study investigated whether stable first- and last-entry behaviour is associated with milk production, milk quality, and udder health in Lacaune dairy ewes. Ewes consistently entering the milking parlour first were generally heavier and calmer than those consistently entering last. They also showed higher milk production in unadjusted comparisons; however, these differences were largely explained by body weight, age, parity, and temperament. In contrast, first-entry ewes showed more favourable udder health indicators, including lower electrical conductivity and fewer udder pathogen-positive milk samples, although bacteriological results should be interpreted cautiously. Milk fat and protein percentages were similar between groups, and differences in fatty acid composition were exploratory. These results suggest that stable milking entry behaviour may serve as a simple, low-cost behavioural indicator reflecting animal characteristics and udder health status in dairy sheep.

## 1. Introduction

In recent decades, the demand for sheep milk and sheep milk products, such as cheese and yoghurt, has increased because of their high nutritional value and greater digestibility compared with cow milk and cow milk products [1,2,3]. Global sheep milk and cheese production exceeded approximately 11 million tons and 511 thousand tons in 2024, respectively [4]. However, expanding the product range and producing premium products for export remain important for the development of sheep milk production. This sector has the potential to become an important branch of animal production by providing employment and marketable products.

Milk composition is known to strongly influence the quality and composition of dairy products. Both the quantity and composition of sheep milk are affected by several factors, including breed, nutrition, and parity. Beyond traditional production-related factors, and in parallel with the intensification of milk production, the evaluation of animal behaviour has become increasingly important. Behavioural traits associated with milking, such as temperament and the order of entry into the milking parlour, may be related to milk production performance. In dairy sheep, behavioural reactivity and temperament have been shown to influence stress responsiveness, milk production, milk ejection, and milking efficiency [5,6,7]. Stress-related responses during machine milking, such as increased leg movements, reflect individual temperament and have also been associated with elevated somatic cell count, linking animal welfare to milk quality [8].

In contrast to temperament, only a limited number of studies have investigated the factors influencing the order of entry into the milking parlour. Margetínová et al. [9] reported that older goats entered the milking parlour earlier than younger goats, whereas Mačuhová et al. [10] found no relationship between parity and entry order. Previous studies suggest that entry order may be associated with milk yield and milkability in dairy ewes, although the traits affected differ among studies. Villagrá et al. [11] reported that later-entering ewes produced less milk and showed poorer milkability, as indicated by a lower proportion of machine milk and a higher proportion of hand-stripped milk. Mačuhová et al. [10] found that entry order was associated mainly with milkability traits, whereas milk composition was not affected. More recent findings also indicate that earlier-entering ewes may have higher milk yield [12]. Nevertheless, no significant correlations have been detected between somatic cell count and the variables studied [11]. Routine behavioural observations during milking have been proposed as non-invasive sources of information on animal responses to the milking environment and may contribute to the interpretation of individual variation in milking-related traits [13,14,15].

However, little is known about whether stable entry behaviour is related to detailed milk quality and udder-health traits in dairy ewes. In particular, information is lacking on physicochemical parameters, hygienic and microbiological indicators, and fatty acid composition. Moreover, because temperament may influence stress responsiveness, milk ejection, and milking efficiency, it should be considered when evaluating associations between entry behaviour and production or udder-health traits.

The aim of this study was to evaluate whether stable first- and last-entry behaviour into the milking parlour is associated with ewe temperament, milk production traits, and milk physicochemical, hygienic, microbiological, and fatty acid parameters. Specifically, the study compared consistent first- and last-entry ewes for body weight, temperament, milk yield, lactation length, milk composition, pH, electrical conductivity, somatic cell count, udder pathogen prevalence, and fatty acid composition.

## 2. Materials and Methods

### 2.1. Experimental Design

This study was carried out on a sheep nucleus farm in Mórichida (47°29′10.5″ N, 17°25′18.4″ E), Győr-Moson-Sopron County, Hungary. A total of 246 Lacaune ewes lambed at the beginning of February. After an 8-week lamb-rearing period, lambs were weaned, and machine milking of the ewes was started. The ewes were between 2 and 6 years of age and had 1 to 5 parities.

The study used an extreme-group design based on repeated observations of milking parlour entry. During lactation, all 246 ewes were monitored for entry order. For the detailed analyses, only ewes that consistently entered either the first or the final milking groups in all six observations were included. This selection was intended to create two clearly defined behavioural groups and to avoid the misclassification of ewes with intermediate or inconsistent entry patterns. Therefore, the analysed animals represent stable first- and last-entry groups rather than the whole flock, and the results should be interpreted as associations within these extreme groups, not as general population-level effects of entry order.

All 246 Lacaune ewes were kept indoors throughout the experimental period and were housed together as a single flock in the same indoor loose-housing pen. The animals had free access to drinking water and were fed the same total mixed ration. No regrouping of the experimental animals was performed during the observation period.

During the milking period, the animals received 100 g of commercial concentrate per milking (NEl: 7.1 MJ/kg dry matter (DM); crude protein: 180 g/kg DM). Feeding was based on a total mixed ration (TMR) consisting of corn silage, alfalfa hay, and concentrate. Commercial trace-mineralized salt blocks containing vitamins A, D3, and E were freely available in the barn. The diets were adjusted according to the National Research Council recommendations for the energy and protein requirements of dairy sheep [16]. The ingredients of the TMR did not change during the study period. The ingredient and chemical composition of the TMR are shown in Table 1. At the beginning of the study, representative samples of the total mixed ration (TMR) were collected and analysed for chemical composition.

Ewes were milked twice daily in a Hungarolact milking system (Agro Legato Ltd., Budapest, Hungary) with 2 × 24 parallel stands at 5:00 a.m. and 5:00 p.m. During milking, animals entered the parlour voluntarily in groups of 2 × 24 individuals. As the parlour contained 48 milking stands, the 246 ewes were milked in five complete groups and one partial group (6th group). Animals spent approximately 5 min in the milking stands, during which they could consume concentrate. Individual milk production was calculated based on monthly milk recording according to the Sheep Performance Testing Codex of the Hungarian Sheep and Goat Breeders’ Association (HSGBA) [17] and the guidelines of the International Committee for Animal Recording [18]. Standard milk yield was defined as the amount of milk produced between days 60 and 150 of lactation [18].

Entry order into the milking parlour was observed on three selected days of lactation: days 46, 74, and 102. These observation days were separated by 28-day intervals and covered the period after the start of machine milking. On each observation day, entry order was recorded during both the morning and evening milkings, resulting in six entry-order observations per ewe. Animals were identified individually by ear tag as they entered the milking parlour. This repeated-observation approach was used to identify ewes with stable entry behaviour under routine milking conditions and to reduce the risk of classifying animals based on a single observation [10,12]. Ewes classified as first-entry ewes were those recorded in the first milking group in all six observations, whereas last-entry ewes were those recorded in the final milking groups in all six observations. Animals with inconsistent or intermediate entry positions were excluded from the detailed group comparison. The final analysed dataset included 18 first-entry ewes and 23 last-entry ewes.

Temperament was assessed at the same six milking observations used for entry-order recording, i.e., during the morning and evening milkings on lactation days 46, 74, and 102. Temperament was scored on a 1 to 5 scale based on ewe behaviour in the milking stand during milking according to Budzynska et al. [19]: 1 = very nervous, continual and vigorous stepping and kicking; 2 = continual and vigorous stepping without kicking; 3 = occasional vigorous leg movements; 4 = quiet standing with few slight leg movements; and 5 = very quiet, no leg movements. Thus, higher temperament scores indicated calmer behaviour. All temperament scoring was performed by the same trained observer to ensure consistency and reduce observer bias.

Prior to individual milk sample collection, the teats were cleaned with antiseptic wipes, and the first three milk jets were discarded. Subsequently, individual milk samples from each udder half were collected aseptically by hand into 10 mL sterile plastic tubes for somatic cell count and bacteriological analysis. After milk yield recording, additional milk samples were collected into 50 mL plastic tubes for the determination of milk chemical composition, fatty acid profile, pH, and electrical conductivity (EC), and were immediately transported to the laboratory.

### 2.2. Milk and Total Mixed Ration Analysis

Representative samples of the total mixed ration (TMR) were analysed for dry matter, crude protein, crude fat, crude fibre, and crude ash according to the procedures of the Hungarian Feed Codex [20].

Milk samples were analysed for the identification of udder pathogen bacterial species. Milk (0.1 mL) was plated on Columbia esculin blood agar (Biolab Inc., Budapest, Hungary) containing 5% sheep blood and 0.5% esculin, and incubated at 37 °C for 48 h. The isolates were identified as udder pathogens by conventional methods, including Gram staining, colony morphology, and hemolysis patterns, according to the Mastitis guidelines [21]. Milk somatic cell count was determined using a LactoScan SCC apparatus (Milkotronic Ltd., Nova Zagora, Bulgaria).

Fat, protein, lactose, and total solids contents of milk were determined using a LactoScope™ IR spectrometry analyzer (Delta Instruments, Drachten, The Netherlands).

Milk pH was measured using an HI98163 pH meter (Hanna Instruments, Carrollton, TX, USA), and electrical conductivity was analysed using a Cond 340i conductivity meter with a WTW Tetracon 325 probe (WTW, Weilheim, Germany).

Milk fat was extracted using the Gerber method [22]. Fatty acids (FAs) were re-esterified to methyl esters according to the ISO 12966-2 (2011) standard [23]. Fatty acid methyl esters were determined by gas chromatography (GC 2010 gas chromatograph, Shimadzu, Kyoto, Japan) with a flame ionization detector (FID) and a Zebron ZB-WAX column (30 m × 0.25 mm × 0.25 μm). The split injection ratio was 50:1. Helium was used as the carrier gas at a flow rate of 28 cm/s. The injector and detector temperatures were 270 and 300 °C, respectively. The oven temperature program started at 80 °C, then increased at 2.5 °C/min to 205 °C and was held for 20 min, after which it was increased again to 225 °C at 10 °C/min and held for 5 min. Peaks were identified on the basis of the retention times of standard methyl esters of individual FAs (Supelco 37 Component FAME Mix, Sigma-Aldrich, St. Louis, MO, USA). Individual FAs were calculated as the ratio of their peak area to the total area of all observed acids. The selected FA groups were calculated using the FA data: saturated fatty acids (SFAs), monounsaturated fatty acids (MUFAs), polyunsaturated fatty acids (PUFAs), total n-6 and n-3 PUFAs, and the n-6/n-3 ratio.

### 2.3. Statistical Analysis

Statistical analyses were performed using IBM SPSS Statistics for Windows, version 27.0 (IBM Corp., Armonk, NY, USA). Prior to analysis, data were checked for completeness, plausibility, and outliers. The normality of data distribution was assessed using the Kolmogorov–Smirnov test, and homogeneity of variances was evaluated using Levene’s test.

Temperament scores were originally recorded on a 1–5 scale. Due to the low number of observations in the two lowest temperament categories, scores 1 and 2 were merged into a single category (1 + 2) before statistical analysis. Temperament scores were compared between the first-entry group (FG) and the last-entry group (LG) using the non-parametric Mann–Whitney U test.

Descriptive statistics were calculated for the main animal-level and production traits. Group differences in body weight, age, parity, production traits, milk physicochemical parameters, somatic cell count, and fatty acid composition were analysed using independent-samples *t*-tests when normality and homogeneity of variance assumptions were met. When these assumptions were not fulfilled, non-parametric tests were applied. Somatic cell count values were log_10_-transformed before analysis and are presented as log_10_ somatic cell count (LSCC).

To quantify the magnitude of unadjusted differences between entry-order groups, Cohen’s d effect sizes were calculated for the main production, milk composition, and udder health traits. Positive values of Cohen’s d indicate higher values in the first-entry group, whereas negative values indicate higher values in the last-entry group. Effect sizes were interpreted as small (*d* = 0.2), medium (*d* = 0.5), and large (*d* ≥ 0.8).

A multivariable logistic regression model was fitted to evaluate the association between entry order and animal-level variables. Entry order was used as the dependent variable, with the last-entry group coded as the outcome category and the first-entry group used as the reference category. Body weight, age, parity, and temperament score were included as explanatory variables. Before interpreting the multivariable model, collinearity among explanatory variables was assessed using correlation analysis, tolerance values, and variance inflation factors (VIF). Particular attention was given to biologically related or potentially associated variables, including age and parity, as well as body weight and temperament score. Odds ratios (OR) and 95% confidence intervals (CI) were calculated. Odds ratios below 1 indicate a lower likelihood of belonging to the last-entry group, whereas odds ratios above 1 indicate a higher likelihood of belonging to the last-entry group.

To account for potential confounding by animal-level characteristics, general linear models were fitted for milk production and udder health traits. These models were based on ewe-level summary values; for repeatedly measured traits, mean values across sampling occasions were first calculated for each ewe. In the primary adjusted models, entry-order group was included as a fixed effect, while body weight, age, parity, and temperament score were included simultaneously as covariates. Because these animal-level variables may be physiologically or behaviourally interrelated, supplementary models were also fitted in which body weight, age, parity, and temperament score were included separately, one at a time, as covariates together with the entry-order group. This approach was used to evaluate whether the association between entry order and the investigated traits depended on the simultaneous inclusion of several potentially related covariates. Adjusted least-square means and standard errors were estimated for the first-entry and last-entry groups, and the significance of the adjusted entry-order effect was evaluated after controlling for these covariates.

For variables measured repeatedly during the study period, mean values across sampling occasions were used for the animal-level summary analyses. Milk fat and protein percentages, as well as fat and protein yields, were also evaluated in relation to repeated sampling structure. Repeated-measures mixed models included entry order group as a fixed effect, sampling time as a repeated factor, and ewe as a random effect. As no significant group × time interactions were detected, only main group effects are presented in the results.

The prevalence of udder pathogen-positive and pathogen-negative milk samples was compared between entry-order groups using the chi-square test. Because bacteriological status was evaluated at the milk-sample level and repeated samples originated from the same animals, these results were interpreted as sample-level associations. The distribution of pathogen categories among infected samples was compared between groups using Fisher’s exact test due to low expected cell counts. Pathogen-specific results are therefore presented descriptively.

For fatty acid composition, *p*-values were first calculated for individual fatty acids and fatty acid groups. To account for multiple testing, *p*-values were adjusted using the Benjamini–Hochberg false discovery rate procedure. Fatty acid results were interpreted primarily on the basis of FDR-adjusted *p*-values and are exploratory.

Results are presented as means ± standard deviation (SD) for unadjusted descriptive and group comparisons, and as adjusted least-square means ± standard error (SE) for the general linear models. Differences were considered statistically significant at *p* < 0.05. All statistical tests were two-sided. Given the small sample size and the use of an extreme-group design, the results of multivariable and exploratory analyses require cautious interpretation. In particular, the logistic regression model should be considered exploratory and potentially prone to overfitting.

Social hierarchy and dominance rank were not directly assessed in the present study; therefore, dominant and subordinate status could not be included in the statistical analyses.

## 3. Results

Descriptive statistics of the analysed ewes according to entry-order classification are summarised in Table 2. The ewes had a mean body weight of 59.20 kg, a mean age of 4.17 years, and an average parity of 2.41. The average temperament score was 3.41. The average lactation length was 154 days, with a mean lactation milk yield of 170.6 kg and an average daily milk yield of 1.11 kg. Mean milk fat and protein contents were 7.69% and 5.72%, respectively, while the average somatic cell count was 5.08 log_10_ cells/mL.

First-entry ewes had higher body weight than last-entry ewes (62.11 ± 5.25 vs. 56.83 ± 4.94 kg, respectively; *p* = 0.002). They also had higher temperament scores, indicating calmer behaviour, than last-entry ewes (4.00 ± 1.08 vs. 2.96 ± 1.26, respectively; *p* = 0.011).

A multivariable logistic regression model was fitted to evaluate the association between entry order and animal-level variables (Table 3). Odds ratios refer to the likelihood of belonging to the last-entry group. In this exploratory model, body weight, age, and temperament showed significant associations with entry-order group, whereas parity was not a significant predictor. Higher body weight and higher temperament scores were associated with a lower likelihood of belonging to the last-entry group, indicating that heavier and calmer ewes were more likely to belong to the first-entry group. In contrast, older age was associated with a higher likelihood of belonging to the last-entry group. Given the limited sample size, these results should be interpreted as exploratory.

Collinearity diagnostics were performed before interpreting the multivariable logistic regression model. Variance inflation factor values ranged from 1.30 to 2.32, and tolerance values ranged from 0.43 to 0.77, indicating no severe multicollinearity among the explanatory variables. Spearman correlation analysis showed a strong positive association between age and parity (*rho* = 0.762, *p* < 0.001), whereas body weight and temperament score were not significantly correlated (rho = 0.122, *p* = 0.448). Therefore, although no severe multicollinearity was detected, the results of the full multivariable model were interpreted cautiously.

Unadjusted comparisons between entry-order groups are presented in Table 4. In addition to statistical significance, effect sizes were calculated to evaluate the magnitude of group differences. The largest effect sizes were observed for lactation milk yield (*d* = 1.31), fat yield (*d* = 1.16), protein yield (*d* = 0.92), and electrical conductivity (*d* = −0.88), indicating moderate to large unadjusted differences between entry order groups. However, these differences require cautious interpretation, as they do not account for animal-level characteristics.

To further evaluate the influence of physiologically related animal-level covariates, supplementary models were fitted in which body weight, age, parity, and temperament score were included separately as covariates together with the entry-order group. In these individually adjusted models, the effect of entry-order group remained significant for lactation milk yield, lactation length, standard milk yield, daily milk yield, fat yield, protein yield, and electrical conductivity. This indicates that none of the individual covariates alone fully explained the unadjusted differences between first- and last-entry ewes.

In the full adjusted model, in which body weight, age, parity, and temperament score were included simultaneously, milk production traits no longer differed significantly between first- and last-entry ewes (Table 5). Thus, the unadjusted production-related differences were attenuated after simultaneous adjustment and should be interpreted cautiously, as they may reflect the combined influence of animal-level characteristics rather than an independent effect of entry order.

In contrast, electrical conductivity remained significantly lower in the first-entry group after full adjustment (*p* = 0.012), suggesting a more consistent association with entry order than the other udder health indicators examined. LSCC was numerically lower in the first-entry group in the unadjusted analysis, but this difference was not statistically significant and remained non-significant after adjustment.

A sample-level difference was observed in the prevalence of udder pathogen-positive milk samples between entry-order groups (Table 6). The proportion of infected samples was lower in the first-entry group (FG; 4.6%) than in the last-entry group (LG; 16.7%; *p* = 0.012). However, because repeated samples originated from the same ewes, these bacteriological results represent exploratory sample-level associations rather than independent animal-level effects. Therefore, statistical significance should be interpreted with caution, as independence assumptions are not fully met.

Within infected samples, coagulase-negative staphylococci (CNS) were the most frequently detected pathogens in the FG, whereas *Escherichia coli*, CNS, and *Streptococcus uberis* were more frequently observed in the LG.

However, the distribution of pathogen categories among infected samples did not differ significantly between groups based on Fisher’s exact test (*p* = 0.107). Therefore, pathogen-specific patterns are descriptive due to the low number of infected samples.

The fatty acid profile of milk samples according to entry order is presented in Table 7. Several fatty acids and fatty acid groups showed nominal differences between groups (*p* < 0.05), including selected saturated and unsaturated fatty acids.

In general, certain short- and medium-chain fatty acids were numerically higher in the first-entry group, whereas some long-chain and monounsaturated fatty acids were higher in the last-entry group. However, after adjustment for multiple comparisons using the Benjamini–Hochberg false discovery rate procedure, none of these differences remained statistically significant. Therefore, these findings are exploratory.

## 4. Discussion

The main finding of the present study is that stable first- and last-entry behaviour into the milking parlour primarily reflected animal-level characteristics and selected udder health indicators, rather than exerting an independent effect on milk production traits. The lactation milk yield (170.6 kg) and lactation length (154 days) of the investigated ewes were more favourable compared with previous reports [24,25,26]. Moreover, the production parameters exceeded the official Hungarian data for Lacaune ewes (lactation milk yield: 145.5 kg; lactation length: 134 days) [27]. The milk fat and protein contents (7.69% and 5.72%) were consistent with earlier studies [25,28,29,30]. Thus, the flock showed milk composition values comparable to those reported for dairy ewes under similar conditions.

Ewes consistently entering the milking parlour in the first group exhibited significantly higher body weight and more favourable temperament scores compared with those entering last. Similar associations between body condition, calm temperament, and priority access to milking facilities have been reported in both sheep and goats [5,6,31]. Heavier and calmer animals may have had greater priority of access to the milking parlour, possibly reflecting differences in social status; however, social rank was not directly measured in the present study. Previous studies also showed that calmer ewes tend to be heavier [32] and produce more milk [6,7]. Higher body weight has likewise been associated with improved milk production [33]. In unadjusted comparisons, the groups did not differ significantly in age or parity. However, the multivariable analysis indicated an association between age and entry order, suggesting a complex and context-dependent relationship between physiological traits and entry behaviour [10]. Although age and parity are biologically related, they did not show identical associations with stable entry order in the present model. This may reflect the limited sample size, the extreme-group design, and the fact that chronological age and reproductive history do not always correspond perfectly in managed dairy flocks. In addition, the simultaneous inclusion of age and parity may have introduced partial collinearity. Therefore, the observed age effect should be interpreted cautiously and confirmed in larger populations.

The relevance of temperament in dairy ewes was already highlighted by Dimitrov-Ivanov and Djorbineva [34], who classified ewes as calm, nervous, or intermediate using a complex behavioural score based on milking-parlour and fear-related tests. They reported that calm ewes produced more milk during machine milking than nervous ewes and that several functional udder parameters differed between temperament groups, with the strongest contrast observed for milk-ejection latency. They also found a significant difference in fertility between calm and nervous ewes at three years of age. These findings are highly relevant to the present study because they indicate that ewe temperament is linked not only to behavioural reactivity during milking, but also to milk removal and udder-related functional traits. In the present study, first-entry ewes were calmer than last-entry ewes, but the adjustment for temperament and other animal-level covariates attenuated the milk-yield differences, suggesting that stable entry behaviour should be interpreted together with temperament rather than as an isolated cause of production differences.

The present results are consistent with earlier studies reporting that animals entering the milking parlour earlier tend to produce higher milk yields [9,10,11]. Villagrá et al. [11] showed that animals entering later often exhibit reduced milk yield and poorer milkability, which may be associated with stress-related inhibition of milk let-down. Similar patterns have been observed in goats, where animals entering first produced consistently higher milk yields throughout lactation [9]. In the present study, higher daily and standardised milk yields were observed in the first-entry group in the unadjusted analysis.

Importantly, after adjustment for body weight, age, parity, and temperament score, the associations between entry order and milk production traits were no longer statistically significant. This indicates that the observed differences in milk yield were largely explained by animal-level characteristics rather than by entry order itself. Therefore, entry order should be interpreted primarily as a behavioural indicator reflecting body weight, temperament, and possibly social rank, rather than as an independent determinant of milk production.

The relationship between milking entry order and milk yield was also described in early cattle studies. Rathore [35] reported that cows showed a relatively consistent order of entry into the milking parlour and that higher-yielding cows tended to enter earlier. This supports the biological plausibility of the unadjusted association observed in the present study, while the adjusted analyses indicate that, in Lacaune ewes, this association was not independent of body weight, age, parity, and temperament. More broadly, classical work on cattle behaviour emphasised that movement through milking facilities and access to resources are shaped by social behaviour, dominance relationships, and motivation [36]. Accordingly, stable first-entry behaviour in the present flock may partly reflect social priority or motivation to access the milking parlour and concentrate offered during milking; however, because social rank was not directly assessed, this interpretation remains hypothetical.

One possible explanation for the observed unadjusted relationships is that delayed access to milking may reflect differences in social stress and milking dynamics, which could potentially influence oxytocin release and milk ejection [37]. However, given the results of the adjusted analyses, it is more likely that entry order captures underlying differences in animal condition and behaviour rather than exerting a direct physiological effect.

Findings from automatic milking systems further support the view that milking-related traffic is a behavioural-management trait rather than a purely production-driven response. Melin et al. [38] showed that cow traffic in an automatic milking system was related to social rank and motivation, and that cows of different ranks may use the waiting area and milking unit differently. More recently, Schwanke et al. [39] demonstrated that individual dairy cow personality traits can modify responses to concentrate allowance in a free-traffic automated milking system, with more fearful cows being less likely to consume the full high concentrate allowance and showing altered intake patterns. Together, these studies underline that entry or traffic behaviour around milking facilities should be interpreted in a broader behavioural and management context. From this perspective, the originality of the present work lies in extending these concepts to dairy ewes by using repeated observations to define stable first- and last-entry behaviour and by combining entry behaviour with temperament, adjusted production analyses, and detailed udder-health, microbiological, and milk-quality indicators.

Daily fat and protein yields were higher in the first-entry group in the unadjusted analysis, which is primarily attributable to their higher milk production. This agrees with previous findings that milk yield is more sensitive to behavioural and management factors than milk composition. In contrast, milk composition is strongly influenced by genetics, nutrition, and stage of lactation [10,28]. The lack of significant differences in fat and protein percentages between groups further supports this interpretation.

A notable finding of the present study is the association between entry order and udder health. Milk from last-entering ewes showed higher electrical conductivity, while LSCC was only numerically higher and did not differ significantly between groups. This pattern suggests that the association between entry order and udder health was more evident for electrical conductivity than for somatic cell count. Electrical conductivity is a well-established indirect indicator of mastitis [40,41,42], and elevated somatic cell counts are commonly associated with intramammary infections. Importantly, after adjustment for animal-level covariates, electrical conductivity remained significantly associated with entry order, whereas LSCC was not significantly associated with entry order in either the unadjusted or adjusted analysis. This suggests that the relationship between entry order and udder health may be more robust for certain indicators than for others.

Udder pathogen prevalence was also higher in the last-entry group. Environmental pathogens such as *Escherichia coli* and *Streptococcus uberis* were numerically more common in this group and are typically associated with hygiene conditions and environmental exposure [43]. However, pathogen-specific patterns are descriptive, as the overall distribution of pathogen categories did not differ significantly between groups. In contrast, coagulase-negative staphylococci (CNS) were the most frequent isolates in the first-entry group. Previous studies reported that CNS are common minor mastitis pathogens and are associated with increased somatic cell counts [44,45].

An alternative explanation for the higher proportion of udder pathogen-positive samples in the last-entry group is the possible effect of milking order itself. Because last-entry ewes were milked later in the milking sequence, they may have been exposed to a greater microbial load in the milking environment or on the milking equipment. This could have increased the probability of exposure to environmental pathogens independently of temperament or stable entry behaviour. This explanation is particularly relevant for environmental pathogens such as *E. coli* and *S. uberis*, whose occurrence may be influenced by hygiene conditions and exposure during milking. Therefore, the higher pathogen prevalence observed in the last-entry group should not be interpreted as a direct consequence of entry behaviour alone. As entry behaviour and milking sequence were closely linked in the present design, their effects cannot be fully separated. Consequently, the bacteriological findings should be interpreted as exploratory associations, and future studies should control for milking sequence, equipment hygiene, and pathogen load during milking.

Udder health and exposure conditions during milking may also contribute to variation in milk fatty acid composition [46]. In the present study, some fatty acid differences appeared to follow the pattern of udder health indicators; however, these results should be interpreted cautiously because the bacteriological findings may have been influenced by milking sequence and environmental exposure. In addition, none of the fatty acid differences remained significant after correction for multiple comparisons. Lower concentrations of short- and medium-chain fatty acids (C4–C14) were observed in the group with higher somatic cell counts. These fatty acids are important for flavour development in dairy products [47]. The proportion of de novo synthesised fatty acids was higher in the group with lower LSCC, which is consistent with the fact that approximately half of milk fatty acids are synthesised de novo in the mammary gland [48]. In contrast, the higher proportion of long-chain fatty acids in the higher LSCC group may be related to altered substrate availability and lipid metabolism [49]. Nevertheless, these findings should be regarded as exploratory.

The observed differences in oleic acid concentration and the oleic-to-palmitic acid ratio may also reflect differences in metabolic status. A higher ratio has been associated with softer milk fat and technological properties relevant to dairy processing [50,51]. However, given the lack of significance after multiple-testing correction, these results require cautious interpretation [52].

Despite the novel insights provided, several limitations of the present study should be acknowledged. The classification of animals into only two extreme categories, first versus last entry, may oversimplify the complexity of entry behaviour, which is likely a continuous and dynamic trait influenced by social interactions and environmental factors. In addition, the small sample size and the selection of only consistently first- and last-entering animals may limit the generalisability of the results. The use of extreme groups may also have amplified the observed differences and potentially inflated effect sizes.

Furthermore, potential confounding factors such as body weight, parity, temperament, and social hierarchy may have contributed to the observed differences between groups. Social hierarchy and dominance rank were not directly assessed in the present study; therefore, it cannot be determined whether stable first- or last-entry behaviour reflected dominance relationships. Another important limitation is that entry behaviour and milking sequence were closely linked, meaning that the effects of stable entry behaviour could not be fully separated from the effects of being milked earlier or later. This is particularly relevant for udder pathogen findings, as exposure to environmental pathogens may have been influenced by milking order, machine contact, and hygiene conditions during milking. Although temperament scoring was performed by the same trained observer and was therefore standardised, observer-related bias cannot be excluded. Finally, given the large number of variables analysed, the risk of type I error cannot be excluded, and some statistically significant findings require cautious interpretation.

Overall, stable entry behaviour into the milking parlour was associated with behavioural traits and selected udder health indicators in dairy sheep. Its relationship with milk production, however, mainly reflected animal-level characteristics rather than an independent entry-order effect. Stable entry behaviour may therefore represent a complementary behavioural observation for identifying animals that could benefit from closer monitoring for handling-related issues or udder health status. However, bacteriological findings should be interpreted cautiously because they may partly reflect milking sequence and environmental exposure rather than entry behaviour alone. Stable entry behaviour should therefore not be used as a standalone diagnostic or management criterion without further validation.

## 5. Conclusions

The present study indicates that stable first- and last-entry behaviour into the milking parlour reflects animal-level characteristics and selected udder health indicators in Lacaune dairy ewes. First-entry ewes were generally heavier and calmer than last-entry ewes. Although first-entry ewes showed higher milk production in unadjusted comparisons, these differences were no longer significant after adjustment for body weight, age, parity, and temperament, indicating that entry behaviour was not an independent determinant of milk production. In contrast, electrical conductivity remained lower in first-entry ewes after adjustment, and udder pathogen-positive samples were less frequent in this group. However, the bacteriological findings should be interpreted cautiously because they may have been influenced by milking sequence and exposure conditions during milking. Overall, stable entry behaviour may serve as a simple complementary behavioural indicator of animal characteristics and udder health status, but it should not be used as a standalone diagnostic or management criterion without further validation in larger studies.

## Figures and Tables

**Table 1:** Ingredient and chemical composition of the total mixed ration (TMR).

Ingredients *	Value
corn silage, %	27.40
alfalfa hay, %	51.52
concentrate, %	21.07
of which:	
winter wheat, %	5.35
corn, %	5.37
rye, %	5.13
oat, %	5.22
Chemical composition	
DM (g/kg fresh matter)	621.30
NEl (MJ/kg DM)	6.17
crude protein (g/kg DM)	140.46
crude fat (g/kg DM)	25.44
crude fibre (g/kg DM)	210.60
crude ash (g/kg DM)	83.36

* Values are expressed on a dry matter basis; DM—dry matter.

**Table 2:** Descriptive statistics for the investigated traits of Lacaune ewes according to entry-order classification.

Trait	First-Entry Group (*n* = 18)	Last-Entry Group (*n* = 23)	*p*-Value
Body weight (kg)	62.11 ± 5.25	56.83 ± 4.94	0.002
Age (years)	3.94 ± 1.47	4.48 ± 1.27	0.231
Parity (n)	2.28 ± 1.60	2.52 ± 1.20	0.517
Temperament score	4.00 ± 1.08	2.96 ± 1.26	0.011
Lactation length (days)	167.78 ± 29.76	143.96 ± 29.22	0.016
Lactation milk yield (kg)	200.31 ± 46.05	147.30 ± 35.37	<0.001
Standard milk yield (kg)	120.92 ± 29.44	101.72 ± 24.44	0.032
Average daily milk yield (kg/day)	1.20 ± 0.22	1.04 ± 0.26	0.046
Fat (%)	7.91 ± 1.09	7.53 ± 1.21	0.320
Protein (%)	5.62 ± 0.54	5.80 ± 0.78	0.430
LSCC (log_10_ cells/mL)	4.93 ± 0.43	5.18 ± 0.65	0.170

Note: Values are presented as mean ± SD. Temperament was scored on a 1–5 scale, where higher values indicate calmer behaviour. LSCC, log_10_-transformed somatic cell count. *p*-values were obtained using independent-samples *t*-tests or Mann–Whitney U tests, depending on distributional assumptions.

**Table 3:** Multivariable logistic regression analysis of factors associated with entry order in Lacaune ewes.

Variable	OR	95% CI	*p*-Value
Body weight (kg)	0.77	0.65–0.92	0.005
Age (years)	2.76	1.06–7.23	0.038
Parity	0.58	0.25–1.34	0.204
Temperament score	0.30	0.12–0.72	0.007

Note: Odds ratios refer to the likelihood of belonging to the last-entry group.

**Table 4:** Milk production traits, physicochemical parameters, log-transformed somatic cell count, and effect sizes of Lacaune ewes according to entry order group.

Trait	First-Entry Group (*n* = 18) Mean	SE	Last-Entry Group (*n* = 23) Mean	SE	*p*-Value	Cohen’s d
Lactation length (days)	167.78	7.01	143.96	6.09	0.016	0.81
Lactation milk yield (kg)	200.31	10.85	147.30	7.38	<0.001	1.31
Standard milk yield (kg)	120.92	6.94	101.72	5.10	0.032	0.72
Daily milk yield (kg/day)	1.20	0.05	1.04	0.05	0.046	0.66
Fat (%)	7.91	0.26	7.53	0.25	0.320	0.33
Fat yield (kg)	16.04	1.14	11.18	0.74	<0.001	1.16
Protein (%)	5.62	0.13	5.80	0.16	0.430	−0.26
Protein yield (kg)	11.28	0.67	8.68	0.59	0.007	0.92
Total solids (%)	18.69	0.36	17.54	0.38	0.104	0.67
pH	6.44	0.03	6.40	0.03	0.133	0.29
EC (mS/cm)	3.66	0.08	4.06	0.11	<0.001	−0.88
LSCC (log_10_ cells/mL)	4.93	0.10	5.18	0.14	0.170	−0.45

Note: Values are presented as ewe-level means ± standard error (SE). For variables measured repeatedly during lactation, mean values were first calculated for each ewe across sampling occasions and then compared between entry-order groups. Somatic cell count values were log_10_-transformed before analysis and are presented as LSCC. *p*-values were obtained using independent-samples *t*-tests or Mann–Whitney U tests, depending on distributional assumptions. Cohen’s d was calculated from ewe-level means using the original group standard deviations. Positive values indicate higher values in the first-entry group, whereas negative values indicate higher values in the last-entry group.

**Table 5:** Adjusted least-square means (±SE) of production and udder health traits according to entry order group.

Trait	First-Entry Group (*n* = 18)	Last-Entry Group (*n* = 23)	*p*-Value
Lactation milk yield (kg)	187 ± 10.3	157 ± 8.8	0.060
Lactation length (days)	162 ± 7.5	148 ± 6.4	0.218
Standard milk yield (kg)	115 ± 7.5	106 ± 6.4	0.426
Daily milk yield (kg/day)	1.17 ± 0.07	1.07 ± 0.06	0.397
Fat yield (kg)	14.5 ± 0.82	12.1 ± 0.70	0.065
Protein yield (kg)	10.5 ± 0.59	8.9 ± 0.51	0.074
Electrical conductivity (mS/cm)	3.72 ± 0.08	4.03 ± 0.07	0.012
LSCC (log_10_ cells/mL)	5.22 ± 0.12	5.36 ± 0.10	0.463

Note: Values are presented as adjusted least-square means ± standard error (SE) obtained from linear models including entry order group as a fixed effect and body weight, age, parity, and temperament score as covariates. *p*-values refer to the adjusted group effect.

**Table 6:** Prevalence and distribution of udder pathogens in milk samples according to entry order group.

Category	First-Entry Group (*n* = 108 Milk Samples)	Last-Entry Group (*n* = 138 Milk Samples)	*p*-Value
Non-infected samples (%)	95.37 (103/108) [90.2–98.3]	83.33 (115/138) [76.1–89.0]	0.012
Infected samples (%)	4.63 (5/108) [1.7–9.8]	16.67 (23/138) [11.0–23.9]	0.012
Distribution within infected samples (%)			0.107
CNS	80.0 (*n* = 4)	30.44 (*n* = 7)	
*Escherichia coli*	0	43.48 (*n* = 10)	
*Streptococcus uberis*	0	17.39 (*n* = 4)	
*Staphylococcus aureus*	20.0 (*n* = 1)	8.69 (*n* = 2)	
Major pathogens (%)	20.0 (*n* = 1)	69.56 (*n* = 16)	

Notes: Values are presented as percentages, with the number of positive or negative samples/total samples in parentheses. For infected and non-infected samples, values in square brackets represent 95% confidence intervals for proportions, calculated using the Wilson method. Differences in the prevalence of infected versus non-infected milk samples between entry-order groups were evaluated using the chi-square test. The distribution of udder pathogen categories among infected samples was compared between groups using Fisher’s exact test due to low expected cell counts; the overall *p* value for pathogen distribution was *p* = 0.107. Individual pathogen categories are presented descriptively only. *n* = number of milk samples. Because repeated milk samples originated from the same ewes, these results represent exploratory sample-level associations.

**Table 7:** Fatty acid profile of milk from Lacaune ewes according to entry order group.

Fatty Acid	First-Entry Group (*n* = 18)	Last-Entry Group (*n* = 23)	*p*-Value	FDR-Adjusted *p*-Value
Lauric acid (C12:0)	3.97 ± 0.81	3.36 ± 0.58	0.010	0.141
Elaidic acid + oleic acid (C18:1)	20.22 ± 2.05	22.67 ± 2.72	0.009	0.141
γ-Linolenic acid	0.04 ± 0.02	0.02 ± 0.01	0.005	0.141
Myristic acid (C14:0)	12.25 ± 1.22	11.20 ± 1.13	0.021	0.187
Docosahexaenoic acid (DHA)	0.02 ± 0.01	0.03 ± 0.01	0.039	0.187
SFAs	73.04 ± 2.37	70.89 ± 3.00	0.038	0.187
MUFAs	22.73 ± 2.33	24.83 ± 2.97	0.040	0.187
Oleic acid/palmitic acid ratio	0.59 ± 0.07	0.67 ± 0.12	0.029	0.187
Fatty acids > C16	36.97 ± 3.60	40.55 ± 4.51	0.024	0.187
Fatty acids < C16	25.97 ± 3.64	23.19 ± 3.08	0.045	0.190
De novo fatty acids	43.22 ± 3.47	40.22 ± 3.68	0.056	0.212
Capric acid (C10:0)	5.98 ± 1.28	5.10 ± 1.06	0.081	0.282
Arachidonic acid	0.22 ± 0.02	0.25 ± 0.04	0.087	0.282
Margaric acid (C17:0)	0.57 ± 0.08	0.61 ± 0.06	0.115	0.345
Conjugated linoleic acid (CLA)	0.49 ± 0.15	0.51 ± 0.10	0.140	0.383
Arachidic acid (C20:0)	0.38 ± 0.05	0.41 ± 0.07	0.155	0.383
Tricosanoic acid (C23:0)	0.04 ± 0.02	0.06 ± 0.03	0.150	0.383
Caprylic acid (C8:0)	1.57 ± 0.36	1.41 ± 0.32	0.245	0.448
Undecanoic acid (C11:0)	0.05 ± 0.02	0.04 ± 0.02	0.197	0.448
Stearic acid (C18:0)	10.45 ± 1.99	11.40 ± 2.27	0.236	0.448
Eicosenoic acid (C20:1)	0.05 ± 0.01	0.05 ± 0.01	0.233	0.448
Eicosapentaenoic acid (EPA)	0.07 ± 0.01	0.06 ± 0.02	0.241	0.448
Behenic acid (C22:0)	0.18 ± 0.03	0.21 ± 0.05	0.206	0.448
Docosapentaenoic acid (DPA)	0.13 ± 0.03	0.15 ± 0.04	0.270	0.472
Tridecanoic acid (C13:0)	0.06 ± 0.01	0.06 ± 0.02	0.293	0.490
n-6/n-3 ratio	2.99 ± 0.35	2.85 ± 0.38	0.303	0.490
Lignoceric acid (C24:0)	0.19 ± 0.05	0.21 ± 0.07	0.361	0.562
Myristoleic acid (C14:1)	0.20 ± 0.08	0.17 ± 0.07	0.443	0.641
Vaccenic acid	1.27 ± 0.27	1.32 ± 0.21	0.430	0.641
Caproic acid (C6:0)	1.28 ± 0.34	1.19 ± 0.37	0.501	0.701
Pentadecanoic acid (C15:0)	0.84 ± 0.11	0.83 ± 0.11	0.678	0.797
Palmitic acid (C16:0)	34.49 ± 2.50	34.06 ± 3.16	0.685	0.797
Palmitoleic acid (C16:1)	1.00 ± 0.32	0.95 ± 0.30	0.683	0.797
α-Linolenic acid	0.72 ± 0.15	0.75 ± 0.23	0.673	0.797
Eicosadienoic acid	0.02 ± 0.01	0.02 ± 0.00	0.718	0.797
Dihomo-γ-linolenic acid	0.02 ± 0.01	0.02 ± 0.01	0.703	0.797
PUFAs	4.23 ± 0.51	4.28 ± 0.76	0.740	0.797
PUFAs n-6	2.80 ± 0.38	2.78 ± 0.54	0.740	0.797
C16 fatty acids	35.49 ± 2.62	35.01 ± 3.32	0.668	0.797
Butyric acid (C4:0)	0.72 ± 0.28	0.76 ± 0.29	0.836	0.877
Linoleic acid	2.50 ± 0.36	2.47 ± 0.51	0.881	0.902
PUFAs n-3	0.95 ± 0.17	1.00 ± 0.26	0.983	0.983

Notes: Values are presented as mean ± SD. *p*-values were obtained using either independent samples *t*-tests or Mann–Whitney U tests, depending on the distribution of the data. FDR-adjusted *p* values were calculated using the Benjamini–Hochberg procedure. SFAs, saturated fatty acids; MUFAs, monounsaturated fatty acids; PUFAs, polyunsaturated fatty acids; EPA, eicosapentaenoic acid; DHA, docosahexaenoic acid; CLA, conjugated linoleic acid.

## Data Availability

The original contributions presented in this study are included in the article. Further inquiries can be directed to the corresponding author.
